# Psychomotor retardation with a 1q42.11–q42.12 deletion

**DOI:** 10.1186/s41065-016-0022-0

**Published:** 2017-03-06

**Authors:** Jialing He, Yingjun Xie, Shu Kong, Wenjun Qiu, Xiaoman Wang, Ding Wang, Xiaofang Sun, Deming Sun

**Affiliations:** 1 Experimental Animal Center, Research Institute for National Health and Family Planning Commission, Tai hui temple road, NO. 12, Haidian District, Beijing, 100081 People’s Republic of China; 2 Key Laboratory for Major Obstetric Diseases of Guangdong Province, Key Laboratory of Reproduction and Genetics of Guangdong Higher Education Institutes, The Third Affiliated Hospital of Guangzhou Medical University, Guangzhou, 510080 China

**Keywords:** 1q42 deletion, Psychomotor retardation, Genotype-phenotype correlation, Microarray analysis

## Abstract

A 1q42 deletion is a rare structure variation that commonly harbours various deletion breakpoints along with diversified phenotypes. In our study, we found a *de novo* 1q42 deletion in a boy who did not have a cleft palate or a congenital diaphragmatic hernia but presented with psychomotor retardation. A 1.9 Mb deletion located within 1q42.11-q42.12 was validated at the molecular cytogenetic level. This is the first report of a 1q42.11-q42.12 deletion in a patient with onlypsychomotor retardation. The precise break points could facilitate the discovery of potential causative genes, such as *LBR, EPHX1*, etc. The correlation between the psychomotor retardation and the underlying genetic factors could not only shed light on the diagnosis of psychomotor retardation at the genetic level but also provide potential therapeutic targets.

## Introduction

Psychomotor retardation has always been described as a slowing of physical and emotional reactions and shared similarities with depression [1]. As a component of depression, psychomotor retardation could provide clinical and therapeutic clues for effective treatments [2]. Specifically, depressed patients were usually classified as melancholic or non-melancholic based on their psychomotor symptoms [3]. Several studies have shown the correlation between psychomotor retardation and depression severity [4, 5]. Furthermore, psychomotor retardation has been speculated to be a potential pathognomonic factor for melancholia [6]. Thus far, a series of indexes has been developed for measuring psychomotor retardation, such as drawing tasks and cognitive, motor, speech and biological tests [7–10]. Although the measurements of psychomotor retardation have been detailed, the underlying genetic pathogenic factors are not well-known.

**Table 1:** Phenotypical comparison of our patient and reported patients with 1q41q42 microdeletion syndrome

Patient/source	Our patient	Rice et al., 2006 [41]	Mazzeu et al.,2010 [42]	Jun et. al.,2013 [43]	Filges et al., 2010 [20]	Decipher 1015	Decipher 266948	Decipher 300673
Coordinate^a^ (chr1:)	224086911-226016203	219978228-225359888	219894313-229156924	223104211-223287570	221885000-227340000	220916999-226162869	222694079-227147000	222821378-226677842
Deletion size	1.9 Mb	5.4 Mb	1 Mb	183 Kb	5.45 Mb	5.25 Mb	4.45 Mb	3.86 Mb
Inheritance/origin	de novo	unknown	De novo	de novo	de novo	de novo	de novo	unknown
Brain Defect	-	+	+	+	+	NR	NR	NR
Cleft Palate	-	+	+	-	+	+	+	+
Hypotonia	-	+	NR	+	-	NR	+	NR
Heart Defect	-	-	+	-	-	+	-	-
Congenital Diaphragmatic Hernia	-	-	-	-	-	+	-	-
Seizures	+	+	-	+	+	-	-	-
Psychomotor Retardation	+	-	-	-	-	-	-	-
Number of Involved Genes^b^	13	>50	>20	2	>50	>40	>60	>50

*NR* No Record

^a^GRCh37/hg19 was used in coordinate

^b^RefSeq genes involved were counted in UCSC browser (http://genome.ucsc.edu/)

Copy number variations (CNVs) have been reported to be associated with dozens of complex diseases, including variant types of cancers, HIV-1/AIDS susceptibility and immunity-related diseases [11–13]. According to previous reports, CNVs usually play an important role in gene dosage, gene disruption, gene fusion, and position effects where abnormal CNVs could cause various diseases [14, 15]. Compared with other deletions, cases with deletion at 1q41–q42 were rarely reported, and existing evidence mostly showed its correlation with congenital diaphragmatic hernia (CDH) and Fryns syndrome [16, 17]. Here, we performed genomic screening using a microarray and discovered a *de novo *1.9 Mb deletion at 1q42.11–q42.12 (chr1: 224,086,911-226,016,203) in a 4-year-old boy showing psychomotor retardation without CDH. Further analysis suggested the involvement of several OMIM (Online Mendelian Inheritance in Man) genes, such as dispatched 1 (*DISP1*) and homo sapiens H2.0-like homeobox (*HLX*). However, *DISP1* and *HLX* always accompanied psychomotor retardation and, thus, were normal in our case.

This study was conducted to refine the clinical presentation of a 1q42.11–q42.12 microdeletion and establish the genotype-phenotype correlation.

### Patient data

The proband was a 4-year-old boy referred to the Clinical Genetics Service for psychomotor retardation. The boy’s parents were unrelated, and both have uneventful family histories. It was reported that the patient was born by normal spontaneous delivery without intrauterine exposure to drugs or other potentially harmful factors. He began speaking single words at the age of 1 year and 8 months and started walking at approximately 2 years old. Mental retardation was observed since the age of 2, and he was diagnosed with psychomotor retardation. In addition, he was risible and particularly friendly to foreigners. A physical examination of the child showed no abnormalities.

## Methods

### Conventional cytogenetic analysis and Fluorescence in Situ Hybridization (FISH)

Peripheral blood samples were collected from three family members with informed consent. A cytogenetic analysis was performed with the standard collection of blood lymphocytes. Metaphase chromosomes were G-banded at 550 bands of resolution.

Metaphase FISH analysis on cultured peripheral blood lymphocytes was performed using a combination of CEP1 (green) probe and single-copy DNA probes (RP11-496N12, 1q42.12, red) that were cloned in BACs (BlueGnome, UK). A minimum of 20 metaphase cells was assessed under a fluorescence microscope (Leica Microsystems, Wetzlar, Germany).

### Chromosomal Microarray Analysis (CMA)–Single Nucleotide Polymorphism (SNP) array analysis

Genomic DNAs were isolated from the peripheral blood samples using a QIAamp DNA Blood Mini Kit (Qiagen, Valencia, CA, USA). The DNA concentrations were measured with a NanoDrop spectrophotometer (ND-1000 V.3.1.2; NanoDrop, Thermo Fisher Scientific Inc., Wilmington, DE, USA). The DNA was amplified, labelled and subjected to 250 ng of product to hybridize CytoScan HD arrays (Affymetrix, Santa Clara, CA, USA) according to the manufacturer’s instructions. The Affymetrix CytoScan HD array covered over 2.7 million markers, of which 750,000 were SNPs that could be used for genotyping, and 1.9 million were non-polymorphic probes. The Chromosome Analysis Suite software package (Affymetrix) was used for all analyses.

## Results

### CMA

**Fig. 1:**
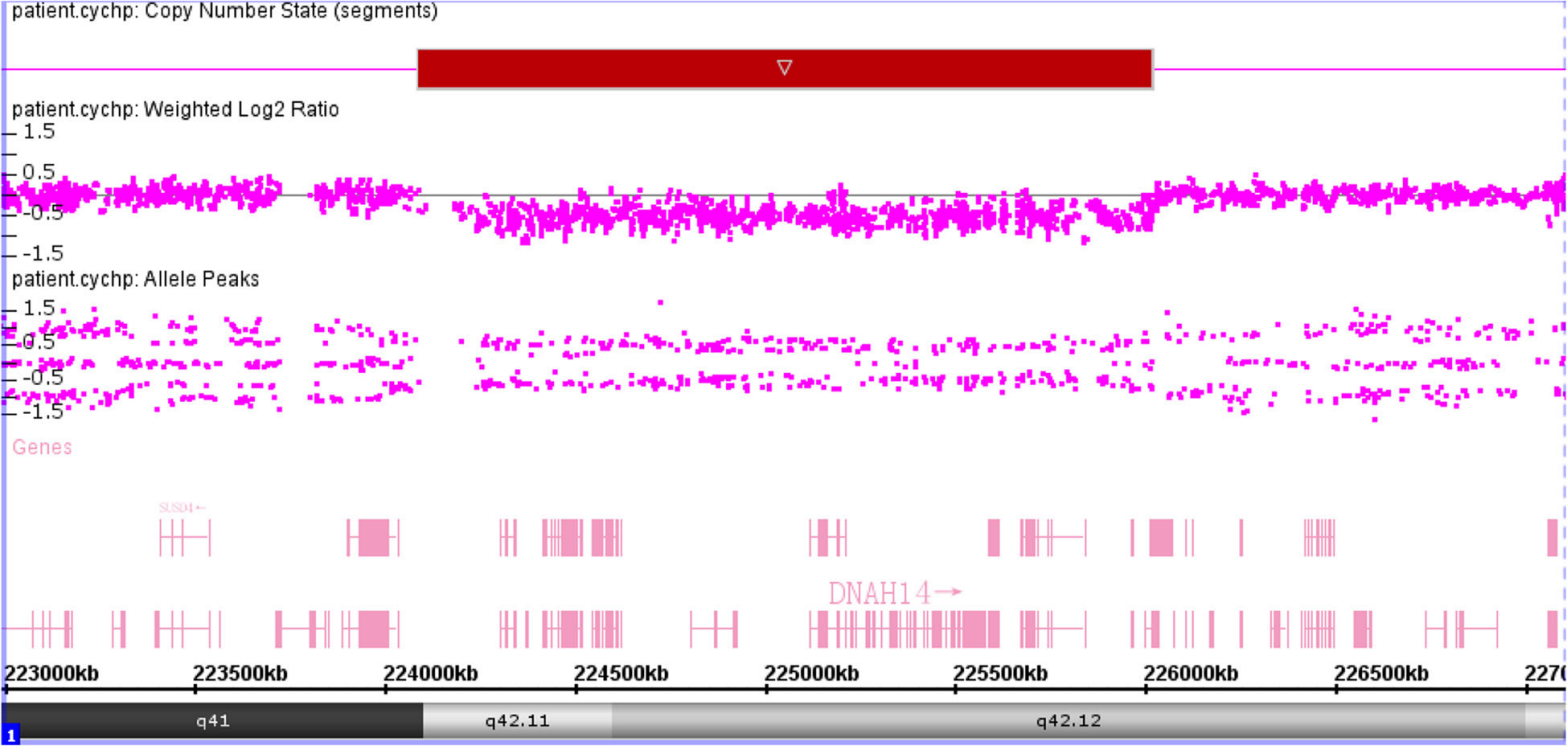
Microdeletion at 1q42.11-q42.12, which spans 1.9 Mb (illustrated with red box in the top)

The genome-wide array analysis of the proband showed a 1.9 Mb deletion at 1q42.11–q42.12 (see Fig. 1), ranging from chr1 as follows: 224,086,911-226,016,203 (GRCh37/hg19). However, his parents showed normal ploidy at the same region.

### FISH and real-time PCR

**Fig. 2:**
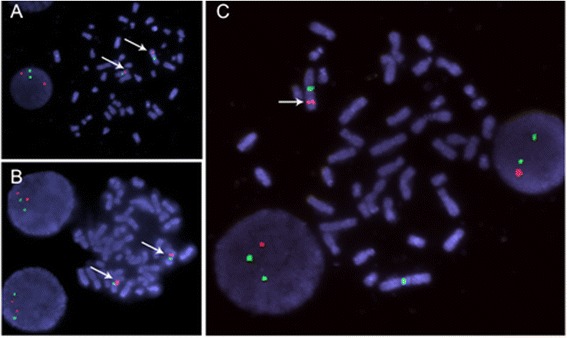
FISH results of cells from the patient and his parents. Two copies of 1q42 were detected in the father (**a**) and mother (**b**), while only 1 copy of 1q42 was retained in the cells of the patient (**c**), thus identifying the deleted region at 1q42. The red fluorescence of 1q42 was indicated with a white arrowhead

The FISH analysis of the parents showed in tegrated 1q42.11-q42.12, while the patient carried only one fragment copy at 1q42.11-q42.12 (see Fig. 2).

**Fig. 3:**
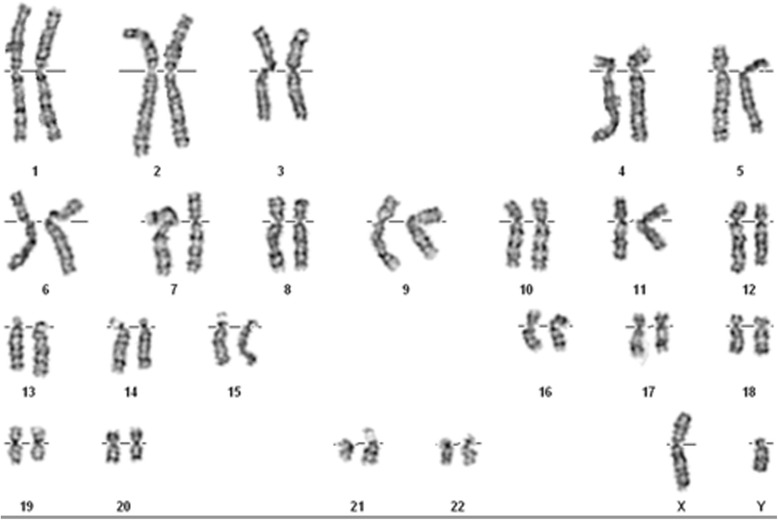
Karyotyping of the cells from the patient. Normal karyotyping was visualized via G-banding techniques with a resolution of 550 bands

In addition, the karyotypes of the parents and the boy were normal (see Fig. 3; data of parents not shown).

## Discussion

Psychomotor retardation could affect physical and emotional reactions and cause speech and walking abnormalities, which are also the most universal manifestations of major depression [1–4]. Furthermore, psychomotor retardation was also involved in adverse effects of drugs, such as benzodiazepines [18]. Since the roles of pathogenesis and important phenotypes in patients with depression remain unclear, the discovery of causative genes is critical. In our studies, the *de novo* 1.9 Mb microdeletion found at 1q42, which was accompanied by 1q41, was mostly reported as the critical region for CDH [17, 19]. However, the data in the Decipher database also suggested connections of common phenotypes in cleft palate, coarse facial features and intellectual disability with a deletion of 1q42.11-q42.12 (Table 1). Nevertheless, neither congenital diaphragmatic hernia nor facial flaw spresenting with signs of psychomotor retardation, such as learning to walk or talk at the age of 2, were observed in our proband. The reason for the sediscrepancies between our case and other reported patients with 1q41q42 microdeletion syndrome is that the deletion in our case only affects the 1.9 Mb spectrum of 1q42 while 1q41q42 deletions of the latter mainly extend into the 1q41 region [17, 20–22].

In the deleted 1.9 Mb range, we found 13 genes, including 7 OMIM genes. Among the seven OMIM genes, most were correlated with development. *FBXO28* is characterized by an approximately 40-amino acid F-box motif and was reported to contribute to intellectual disability, seizures and a dysmorphological phenotype in patients with 1q41q42 microdeletion syndrome [23, 24]. At the molecular level, *FBXO28* could act as a master regulator of cellular homeostasis by targeting key proteins for ubiquitination. For example, *FBXO28* could mediate the degradation of Alcat1 via targeting Alcat1 for monoubiquitination at K183. Meanwhile, *FBXO28* could also function inubiquitylation-independent pathways, including the transmission of *CDK* activity to MYC function during the cell cycle [25, 26]. Nevertheless, more studies are needed to elaborate the detailed molecular pathogenic mechanism of *FBXO28* in 1q41q42 syndrome. *NVL*, known as nuclear *VPC* (valosin containing protein)/p97-Like, is another OMIM gene belonging to the AAA-ATPase (ATPases associated with various cellular activities) super family. The major isoform of *NVL* is NVL2, which was mainly localized in the nucleus and participated in ribosome biosynthesis [27–29]. Wang et al have investigated 1045 major depressive disorder patients, 1235 schizophrenia patients and 1235 normal controls of Han Chinese origin and found that the *NVL* gene could confer risks for both major depressive disorder and schizophrenia in the Han Chinese population [30]. The high correlation between *NVL* and major depression suggested that *NVL* was a potential causative gene for psychomotor retardation. As a gene encoding an axonemal dynein heavy chain, the deletion of *DNAH14* was associated with motile cilia function. Although *DNAH14* is an important gene for motile cilia, further research is needed to understand its contribution to psychomotor retardation. *SRP9* encodes a 9k Da signal recognition particle. A *SRP9* and *RP14* complex was reported to be involved in the elongation arrest function of SRP, which was important to the co translational targeting of secretory and membrane proteins to the endoplasmic reticulum (ER) [31]. Furthermore, *SRP9* also showed higher expression levels in human colorectal cancer [32]. To date, the importance of the role of *SRP9* in its contribution to psychomotor retardation is not well-studied. *LBR* and *EPHX1* genes are located in regions that are frequently deleted in 1q42.11q42.12 deletion syndrome and thus should be investigated because of their neuronal significance. *LBR* encoded lamin B receptor which belongs to ERG4/ERG24 family and was also shown to be a pivotal architectural protein that plays an important role in the nuclear envelope [33, 34]. Mutations in the *LBR* gene could also affect neutrophil segmentation and sterol reeducates activity. *LBR* was associated with two different recognized clinical conditions, Pelger-Huet anomaly (PHA) and Greenberg skeletal dysplasia [35, 36]. On the other hand, *GravemannS*. et al. have shown that the copy number of *LBR* and nuclear segmentation index of neutrophils were highly correlated while the gene-dosage could affect granulopoiesis [37]. Recently, Mc Caffery JMand colleagues have found that the two SNPs (rs2230419 and rs1011319) in LBR were associated with baseline Beck Depression Inventory scores, which also suggested the potential role of the gene in depression. *EPHX1* gene encoded epoxide hydrolase, a critical biotransformation enzyme that converted epoxides to trans-dihydrodiols that could be conjugated and excreted from the body [38]. The dysfunction of *EPHX1* was reported to contribute to several human diseases, including neurodegeneration where its differential expression was presented in patients with Alzheimer’s disease [39]. Additionally, *EPHX1* affected the cerebral metabolism of epoxyeicosatrienoic acids and, hence regulated neuronal signal transmission in mice [40]. Although there is no direct evidence on the causal relationship between *EPHX1* and psychomotor retardation, studies have revealed the important function of *EPHX1* in neuron systems and suggested the potential role of *EPHX1* in neuronal development, which if dysfunctional could lead to psychomotor retardation.

The most common midline defects of 1q42.11–q42.12 deletion syndrome are cleft palate and CDH. However, these manifestations did not appear in our patients. Compared with patients with a cleft palate and CDH, the 1.9 Mb deletion region in our case did not cover *DISP1* and *Shh*, which were reported to be involved in the pathogenesis of developmental defects and CDH [16, 20]. The normality of *DISP1* and *Shh* seems to be the major reason for the absence of a cleft palate and CHD in our patient.

The complex intra chromosomal gene interactions and positional effects are of great importance in complex patterns of midline defects and genes involved in developmental pathways. Our study of a 1q42.11–q42.12 deletion in a boy without a cleft palate but with psychomotor retardation has provided evidence regarding the genotype-phenotype correlation between a 1q42.11–q42.12 microdeletion and psychomotor retardation; especially important is the finding of potential causative genes, such as *LBR* and *EPHX1*, which could become therapeutic targets. Nonetheless, more studies are needed to explore the detailed molecular mechanism of psychomotor retardation pathogenesis.

## Abbreviations

CNVs: Copy number variations
CDH: Congenital diaphragmatic hernia
DISP1: Dispatched 1
HLX: H2.0-like homeobox
SNP: Single nucleotide polymorphism
FISH: Fluorescence in situ hybridization
CMA: Chromosomal microarray analysis
OMIM: Online mendelian inheritance in man
PHA: Pelger-Huet anomaly
